# Hyperchloremia in Intensive Care Unit Mortality: An Underestimated Fact

**DOI:** 10.7759/cureus.4770

**Published:** 2019-05-28

**Authors:** Zahid Siddique Shad, Muhammad Shoaib Safdar Qureshi, Aayesha Qadeer, Azmat Abdullah, Kamran Munawar, Muhammad Tariq Khan, Luqman Saeed, Syed Waqar Hussain

**Affiliations:** 1 Internal Medicine, Shifa International Hospital, Islamabad, PAK; 2 Internal Medicine, Khan Research Laboratories Hospital, Islamabad, PAK

**Keywords:** hyperchloremia, icu

## Abstract

Objective

The goal of the study was to determine the percentage of hyperchloremia in patients who died in medical intensive care unit (ICU) and thus emphasizing the need of avoiding chloride-rich solutions due to their deleterious effects.

Study design

We conducted a retrospective study of data from 206 patients who expired in medical ICU in one year from January 2017 to December 2017 in the department of critical care medicine at Shifa International Hospital, Islamabad.

Material and methods

The study included 206 patients: 93 (43.1%) men and 123 (56.9%) women, over the age of 18 years who expired in medical ICU in one year from January 2017 to December 2017. Patients included for the study were all those who expired with any diagnosis but those who remained admitted in ICU for at least 72 hours and received intravenous fluids. The serum chloride levels of the patients at the time of admission and at 72 hours of stay in ICU were collected. The patients who were having serum chloride levels of 107 milliequivalent per deciliter (meq/dl) or more were labeled as having hyperchloremia. The data were analyzed using IBM SPSS Statistics for Windows, Version 23.0 (IBM Crop., Armonk, NY, USA). The mean and standard deviations were calculated for continuous variables while frequency and percentages were calculated for qualitative variables.

Results

Among 206 patients who expired in our ICU, 109 (50.5%) patients had hyperchloremia at 72 hours of admission in ICU while 107 (49.5%) patients did not had hyperchloremia. Hyperchloremia was more frequent in patients with sepsis or septic shock.

Conclusion

Higher percentage (50.5%) of hyperchloremia at 72 hours of admission among patients (who expired in our medical ICU) indicates excessive use of chloride-rich intravenous fluids. This finding may have significant impact on mortality along with other contributing factors that lead to death of the patients. Keeping in view the findings of the study, chloride-rich solutions should be used carefully to counter the effects of hyperchloremia in patients requiring large volume fluid resuscitation in ICU. Fluids with lower content of chloride such as lactated ringer may be equally good in large volume fluid resuscitation with advantage of avoiding hyperchloremia.

## Introduction

Chloride is one of the most important and the most abundant anion in the extracellular fluid contributing approximately one-third extracellular fluid tonicity [1]. Chloride plays several physiologically important roles in the body which include acid-base balance, immune modulation, osmosis and muscular activity [2]. Acid base homeostasis is the main function, chloride plays in the body. Chloride levels are inversely related to bicarbonate which is thought to be the major buffer in human body. So, increasing or decreasing levels of chloride have direct impact on acid base status, resulting in non-anion gap metabolic acidosis and metabolic alkalosis, respectively.

In gastrointestinal tract, chloride has got two unique functions: first, it is secreted as a part of hydrochloric acid in the stomach and helps in protein digestion, keeping a check on microorganism and absorption of some of the important nutrients/minerals [2]; second, it maintains the gastrointestinal osmotic gradient and fluid secretion. Other effects of chloride are on unloading of oxygen from hemoglobin, immune modulation and cardiovascular stability.

Serum chloride levels are maintained in normal range mainly by the kidneys and gastrointestinal system. Dyschloremia in intensive care unit (ICU) is a common observation in patients who are receiving intravenous fluids or diuretics. Hyperchloremia is somewhat more observed as compared to hypochloremia in ICU patient. Despite its physiologically important functions, chloride has caught little or no attention by the researchers until recently [3] when association of chloride-rich solutions to hyperchloremia metabolic acidosis has been established [4, 5]. It has been found that hyperchloremia increases short-term mortality after non-cardiac surgery [6, 7].

Normal saline (0.9% saline) is the chloride-rich solution most commonly used in clinical practice especially in critically ill patients and preoperatively. Contrary to the name, 0.9% saline in reality is not normal or neutral solution [8]. It has got chloride in supra physiological amount when we compare it with plasma (154 vs. 100 meq/L, respectively) [9, 10]. The liberal use of such a chloride-rich solution results in hyperchloremic metabolic acidosis in critically ill patients [11, 12] with several detrimental effects [13, 14] especially in patients with severe sepsis and septic shock [15]. Hyperchloremia has been associated with acute kidney injury in ICU patients and likewise severe acidosis when present can result in decreased efficacy of inotropes as well as cardiovascular dysfunction. All these deleterious effects of hyperchloremia increase in mortality. Septic patients in critical condition are commonly exposed to 0.9% saline during the salvage phase of septic shock and are at risk of hyperchloremic metabolic acidosis and other detrimental effects of hyperchloremia in the post-resuscitation phase. Observational studies evaluating the association of hyperchloremia with hospital mortality have shown conflicting results and included only a small number of septic patients in the ICU [16-18].

In our ICU, we commonly use normal saline to resuscitate patients. The purpose of the study was to find in what percentage of expired patients hyperchloremia is present so that we may change our practice of using chloride-rich solution and prefer solution with acceptable levels of chloride (lactated ringer or plasmalyte) to avoid this iatrogenic complication which may benefit our patients in future.

## Materials and methods

We conducted a retrospective review of patients’ medical records at the department of critical care medicine at Shifa International Hospital Islamabad. The ethical review board of Shifa International Hospital Islamabad provided ethical approval for the study. We reviewed mortality data in ICU from January 2017 to December 2017. The inclusion criteria entitled all patients eligible who expired in ICU due to any disease/diagnosis in the specified duration of study but an essential stay in ICU for at least 72 hours before expiration and receiving intravenous fluids was a must. Two hundred and six patients (93 men and 123 women) with age range 18 years to 100 years met our inclusion criteria. Patients were categorized into two groups depending on the diagnosis: group 1, patients with diagnosis of sepsis or septic shock and group 2, patients with diagnosis of other than sepsis. Serum chloride levels of the patients were recorded at the time of admission in ICU and then at 72 hours of admission in ICU. A serum chloride level of 107 meq/dl or more was labeled as hyperchloremia. The data were analyzed using IBM SPSS Statistics for Windows, Version 23.0 (IBM Crop., Armonk, NY, USA). The mean and standard deviations were calculated for continuous variables while frequency and percentages were calculated for qualitative variables. Cross tabulation was done for diagnosis and hyperchloremia.

## Results

Two hundred and six patients met the inclusion criteria for the research. Of these 93 (43.1%) were male and 123 (56.9%) were female. The minimum age was 18 years and maximum age was 100 years. The mean age of the patients was 60.62 years with standard deviation of 14.784. Mean chloride level of the patients at the time of admission in ICU was 99.796 meq/dl and mean chloride level of the patients at 72 hours of admission in ICU was 107.5093 meq/dl. We divided the patients, on the basis of diagnosis, into two groups: 1) Sepsis and septic shock group, and 2) diagnosis other than sepsis. One hundred and nineteen patients were in sepsis and septic shock group and 97 patients were in group with diagnosis other than sepsis. All these patients received intravenous fluids. Hyperchloremia was present in 109 (50.5%) patients and 107 (49.5%) patients were not having hyperchloremia at 72 hours of ICU admission. Hyperchloremia was present among 97 patients out of 119 patients in sepsis/septic shock group and 12 patients out of 85 in patients with diagnosis other than sepsis. Among men 50 (53.76%) out of 93 developed hyperchloremia and among women 59 (47.96%) out of 123 developed hyperchloremia at 72 hours of ICU admission. The detailed results are shown in Tables 1-6.

**Table 1:** Descriptive statistics for age, chloride level at admission and chloride levels at 72 hours.

	N	Minimum	Maximum	Mean	Std. Deviation
Age	216	18	100	60.62	14.784
Chloride level at admission	216	84.00	115.00	99.7963	3.99362
Chloride levels at 72 hours	216	92.00	119.00	107.5093	5.86514
Valid N (listwise)	216				

**Table 2:** Chloride levels at admission and at 72 hours.

	N	Minimum	Maximum	Mean	Std. Deviation
Age	216	18	100	60.62	14.784
Chloride level at admission	216	84.00	115.00	99.7963	3.99362
Chloride levels at 72 hours	216	92.00	119.00	107.5093	5.86514
Valid N (listwise)	216				

**Table 3:** Frequency of hyperchloremia among patients at 72 hours of admission.

		Frequency	Percent	Valid Percent	Cumulative Percent
Valid	Hyperchloremia present	109	50.5	50.5	50.5
	Hyperchloremia not present	107	49.5	49.5	100.0
	Total	216	100.0	100.0	

**Table 4:** Frequency of gender of the patients.

		Frequency	Percent	Valid Percent	Cumulative Percent
Valid	Male	93	43.1	43.1	43.1
	Female	123	56.9	56.9	100.0
	Total	216	100.0	100.0	

**Table 5:** Hyperchloremia and diagnosis--cross tabulation.

		Diagnosis		Total
		Sepsis/septic shock	Other than sepsis	
Hyperchloremia	Hyperchloremia present	97	12	109
	Hyperchloremia not present	22	85	107
Total		119	97	216

**Table 6:** Hyperchloremia and gender of the patient–cross tabulation.

		Gender		Total
		Male	Female	
Hyperchloremia	Hyperchloremia present	50	59	109
	Hyperchloremia not present	43	64	107
Total		93	123	216

## Discussion

Chloride is one of the most important and the most abundant anion in the extracellular fluid contributing approximately one-third extracellular fluid tonicity [1]. Chloride plays several physiologically important roles in the body which include acid-base balance, immune modulation, osmosis and muscular activity [2]. Acid base homeostasis is the main function chloride plays in the body. Large volume fluid resuscitation is common in ICU and selection of fluid type is very important in this regard. If chloride-rich solutions are used for large volume resuscitation in septic patients, it may result in hyperchloremia and its untoward effects.

A number of studies have pinpointed the hazardous effects of hyperchloremia in critically ill patients. In a large retrospective study by Neyra et al., deleterious effects of hyperchloremia in ICU patients were studied. The study included 1940 patients. Of these 615 (31.7%) patients had hyperchloremia on ICU admission and 1325 (68.3%) patients were having no hyperchloremia at the time of admission in ICU. Patients were observed from the time of admission in ICU until discharge from hospital or death. During observation period, 431 (23.9%) patients died that included 147 (23.9%) patient in subgroup of hyperchloremia and 284 (21.4%) patients in subgroup without hyperchloremia. This significant mortality difference in both groups suggests hyperchloremia increases mortality in ICU patients. Moreover, patients in hyperchloremia subgroup required more vasopressors support, higher number of blood transfusions, prolonged stay in ICU due to more days on mechanical ventilation and these patients were having more frequent oliguria. These observations along with other studies signify the importance of maintaining physiological range of serum chloride in ICU patients [19].

A large, cluster-randomized, multiple-crossover trial conducted by Semler et al., compared normal saline and balanced crystalloids (lactated ringer or plasma lyte A) for fluid resuscitation in ICU. In this research, 15802 patients in five ICUs were randomized to receive either normal saline (0.9% sodium chloride solution) or balanced crystalloids fluids (lactated Ringer’s solution or Plasma-Lyte A). The primary outcome of the study was to find a major adverse kidney event within 30 days, new renal replacement therapy (RRT) or persistent renal dysfunction defined as elevation of serum creatinine level to ≥200% of baseline or death from any cause. All information were analyzed at the time of discharge/death or 30 days whichever came first. The results were in favor of subgroup with balanced crystalloid administration versus normal saline. Among the 7942 patients in balanced-crystalloid group, 14.3% (1139 patients) had a major kidney-related event while in the normal saline group the percentage of the same was 15.4% (1211 out of 7860 patients). Considering other parameters, 30 days mortality in hospital was 10.3% in the balanced saline group and 11.1% in the saline group (p-value 0.06). The incident of new renal replacement therapy was 2.5% in the balanced saline group and 2.9% in the normal saline group (p-value 0.08) and the incidence of persistent renal dysfunction was 6.4% and 6.6% (p-value 0.60), respectively. The researchers concluded that balanced crystalloid solution has an edge over normal saline in terms of causing less organ dysfunction [20].

In our study, we reviewed the mortality data of those patients who remained in ICU for at least 72 hours before expiration and essentially received intravenous fluids during the stay. The fluid was normal saline exclusively. The purpose was to find how many patients developed hyperchloremia at 72 hours of stay in ICU while receiving intravenous fluids. The study results were astonishing: a significantly higher percentage of hyperchloremia (50.5%) was noted among patients at 72 hours of stay in ICU. It signifies that we are overusing chloride-rich solution: almost exclusively 0.9% saline which has supra physiological content of chloride that can lead to dose dependent hyperchloremic metabolic acidosis and other hazards. Reviewing the available literature on effects of hyperchloremia in critically ill patients, the situation seems alarming and emphasizes that we have to be careful in selection of fluid while we need to use large volume fluid resuscitation in our patients. One more interesting aspect of the study result was lower frequency of hyperchloremia in patients who were having diagnosis other than sepsis that included all other patients except sepsis or septic shock. The reason was overall less fluid administration when compared to the group with diagnosis of sepsis.

## Conclusions

Keeping in view the findings of the study, chloride-rich solutions should be used carefully to counter the effects of hyperchloremia in patients requiring large volume fluid resuscitation in ICU. Balanced crystalloids (lactated ringer or plasmalyte) are equally good choices for large volume resuscitations in critically ill patients with lower risk of hyperchloremia and its effects. The study has some weaknesses as regard to relation of hyperchloremia and total amount of fluids administered. Yet the finding of higher frequency of hyperchloremia in patients who died in ICU clearly advocates using intravenous solutions with low content of chloride to avoid the evitable iatrogenic adverse effect of fluid resuscitation when appropriate.
